# Transanal endoscopic microsurgery versus radical resection for early-stage rectal cancer: a systematic review and meta- analysis

**DOI:** 10.1007/s00384-023-04341-9

**Published:** 2023-02-17

**Authors:** Wei Li, Xing Xing Xiang, Hong Da Wang, Chen Jun Cai, Ying Hao Cao, Tao Liu

**Affiliations:** 1 Department of Pancreatic Surgery, Union Hospital, Tongji Medical College, Huazhong University of Science and Technology, Wuhan , Hubei Province, 430022 China; 2 Department of Trauma Surgery, Union Hospital, Tongji Medical College, Huazhong University of Science and Technology, Wuhan , Hubei Province, 430022 China; 3 Cancer Center, Union Hospital, Tongji Medical College, Huazhong University of Science and Technology, Wuhan, 430022 China

**Keywords:** Early-stage rectal cancer, Transanal endoscopic microsurgery, Radical surgery, Total mesorectal excision

## Abstract

**Purpose:**

In the treatment of early-stage rectal cancer, a growing number of studies have shown that transanal endoscopic microsurgery is one of the alternatives to radical surgery adhering to total mesorectal excision that can reduce the incidence of adverse events without compromising treatment outcomes. The purpose of this meta-analysis is to compare the safety and treatment effect of transanal endoscopic microsurgery and radical surgery adhering to total mesorectal excision to provide a basis for clinical treatment selections.

**Method:**

We searched the literatures of four major databases, PubMed, Embase, Web of science, and Cochrane Library, without limitation of time. The literatures included randomized controlled studies and cohort studies comparing two surgical procedures of transanal endoscopic microsurgery and radical surgery adhering to total mesorectal excision. Treatment effectiveness and safety results of transanal endoscopic microsurgery and radical surgery were extracted from the included literatures and statistically analyzed using RevMan5.4 and stata17.

**Result:**

Ultimately, 13 papers were included in the study including 5 randomized controlled studies and 8 cohort studies. The results of the meta-analysis showed that the treatment effect and safety of both transanal endoscopic microsurgery and radical surgery in distant metastasis (RR, 0.59 (0.34, 1.02), *P* > 0.05), overall recurrence (RR, 1.49 (0.96, 2.31), *P* > 0.05), disease-specific-survival (RR, 0.74 (0.09, 1.57), *P* > 0.05), dehiscence of the sutureline or anastomosis leakage (RR, 0.57 (0.30, 1.06), *P* > 0.05), postoperative bleeding (RR, 0.47 (0.22, 0.99), *P* > 0.05), and pneumonia (RR, 0.37, (0.10, 1.40), *P* > 0.05) were not significantly different. However, they differ significantly in perioperative mortality (RR, 0.26 (0.07, 0.93, *P* < 0.05)), local recurrence (RR, 2.51 (1.53, 4.21), *P* < 0.05),_overall survival_ (RR, 0.88 (0.74, 1.00), *P* < 0.05), disease-free-survival (RR, 1.08 (0.97, 1.19), *P* < 0.05), temporary stoma (RR, 0.05 (0.01, 0.20), *P* < 0.05), permanent stoma (RR, 0.16 (0.08, 0.33), *P* < 0.05), postoperative complications (RR, 0.35 (0.21, 0.59), *P* < 0.05), rectal pain (RR, 1.47 (1.11, 1.95), *P* < 0.05), operation time (RR, −97.14 (−115.81, −78.47), *P* < 0.05), blood loss (RR, −315.52 (−472.47, −158.57), *P* < 0.05), and time of hospitalization (RR, −8.82 (−10.38, −7.26), *P* < 0.05).

**Conclusion:**

Transanal endoscopic microsurgery seems to be one of the alternatives to radical surgery for early-stage rectal cancer, but more high-quality clinical studies are needed to provide a reliable basis.

## Introduction

Worldwide, colorectal cancer ranks third and second in terms of incidence and mortality of all tumors, respectively, posing a serious threat to human life and health [1].

With the improvement of health awareness and medical treatment, an incraeasing number of patients of rectal cancer can be diagnosed at early stage, but the choice of surgical procedure for early rectal cancer is still controversial. Radical surgery following the principles of total mesorectal excision has been the standard procedure for early-stage rectal cancer, which was promoted by professor Richard (Bill) Heald and is being embraced by more clinicians and patients. The scope of total mesorectal excision includes the rectum, rectal mesentery, and occult metastatic lymph nodes. Five-year cancer-specific survival is expected to exceed 95% [2, 3]. Although radical surgery adhering to total mesorectal excision is effective in removing tumors and reducing tumor recurrence rate, there is still a 2% mortality rate and a significant risk of local recurrence [4, 5]. At the same time, postoperative complications such as bowel dysfunction, urinary incontinence, sexual dysfunction, and temporary or permanent stoma occur due to excessive surgical excision, which seriously impairs the quality of life [6–8]. Therefore, with the development of medical technology, transanal endoscopic microsurgery has received more attention. In 1983, Buess et al. introduced transanal endoscopic microsurgery, which is usually used for T_1_ or T_2_ without lymphovascular invasion and distant metastasis. Transanal endoscopic microsurgery (TEM) is an advanced surgical procedure performed through the anus to remove polyps and early cancers from the rectum. The surgery is minimally invasive. The advantages of transanal endoscopic microsurgery include shorter hospitalization time, less intraoperative bleeding, shorter operative time, higher rectum preservation rate, preserving anal function, lower perioperative mortality rate, and lower postoperative complication rate [9–11]. Transanal endoscopic surgery has been shown to be more effective in terms of reducing the incidence of postoperative complications. In early-stage rectal cancer, the probability of lymph node metastasis is 8.6% [9]. However, transanal endoscopic microsurgery cannot remove occult metastatic lymph nodes and may obtain positive margins, which in turn leads to a higher rate of local recurrence [12].

**Fig. 1:**
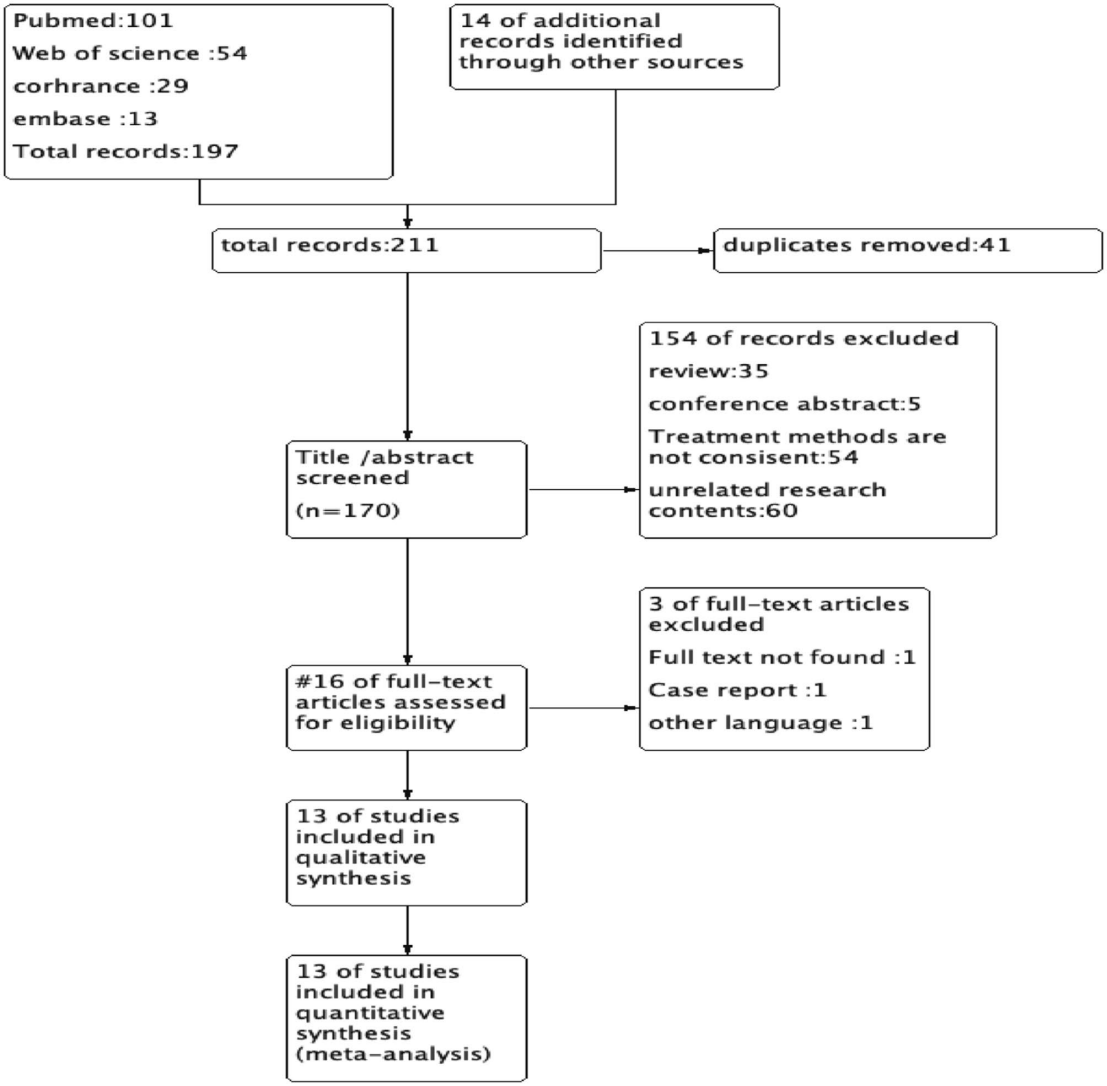
Literature screening flowchart

**Fig. 2:**
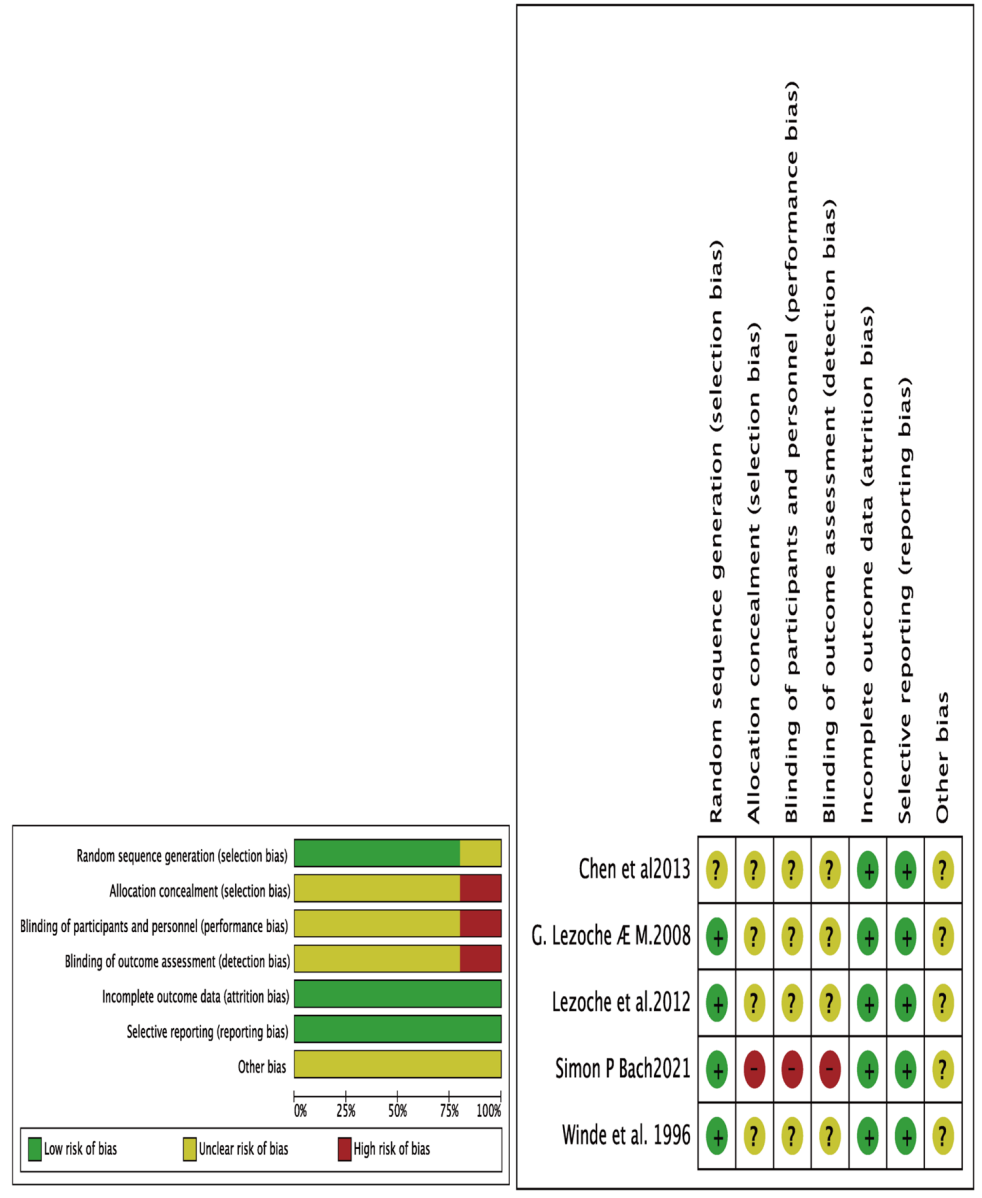
Risk of bias assessment for randomized controlled trials. **A** Weighted bar graph of the distribution of bias risk judgments within each bias domain **B**

**Fig. 3:**
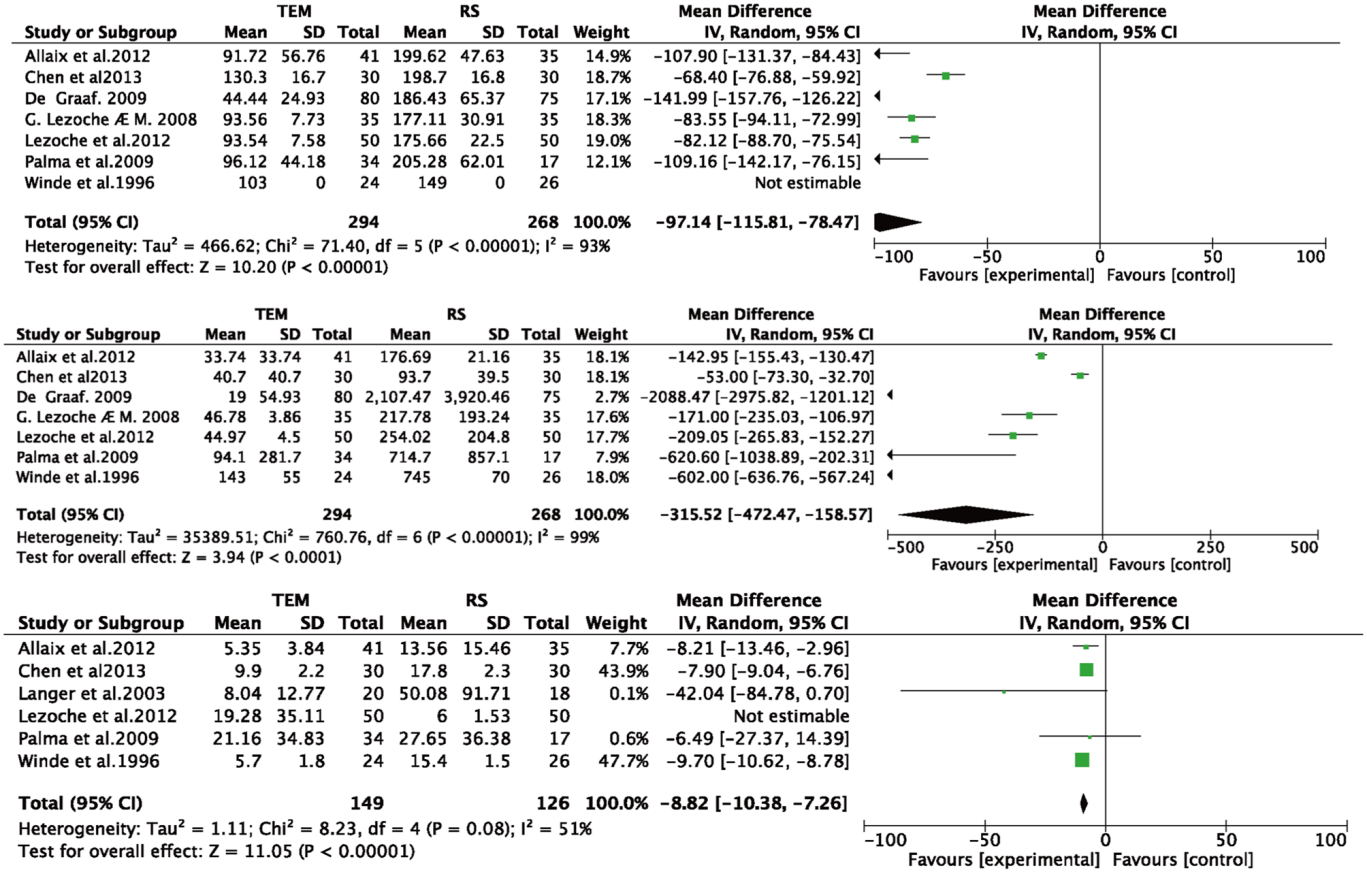
Forest plot **A**. Operative time **B**. Blood loss **C**. Time of hospitalization

Because of the advantages and disadvantages of both surgical approaches, there is uncertainty in the choice of surgical approach for patients with early-stage rectal cancer. Therefore, the purpose of this meta-analysis is to compare the safety and short-term or long-term outcomes of the two surgical approaches to provide a basis for treatment decisions in clinical practice Figs. 1, 2 and 3.

## Methods

### Search strategy and study selection

To conduct this meta-analysis, we searched PubMed, Embase, Web of Science, and Cochrane Library four major English databases, with language of the publications limited to English and publication time unrestricted and with a combination of subject terms and free words to develop a search strategy; in order to obtain all literatures related to the selected topic, the search strategy is shown in Table 1. Two researchers independently read the titles and abstracts of the retrieved literatures. Duplicate studies, conference abstracts, reviews, animal experiments, inconsistent interventions, and irrelevant research content have been excluded. And randomized controlled studies and cohort studies including interventions for patients of early rectal cancer with TEM and radical surgery adhering to total mesorectal excision have been included. After the initial screening, the full text was read to determine the included literature. Those which could not access the full text would also be excluded. Finally, the two investigators reached a consensus through discussion to determine the included literature. The two investigators extracted the perioperative indicators and tumor outcomes after the two interventions of TEM and RS, radical surgery adhering to total mesorectal excision.

**Table 1:** Search strategy

Data			August 2022		
Database	PubMed	Web of Science		Embase	Cochrane library
Search strategy					
Early Stage Rectal Neoplasms OR Early Stage Neoplasm, Rectal OR Early Stage Rectal Neoplasm OR Early Stage Rectum Neoplasms OR Early Stage Neoplasm, Rectum OR early stage Rectum Neoplasm OR early stage Rectal Tumors OR early stage Rectal Tumor OR early stage Tumor, Rectal OR early stage Neoplasms, Rectal OR early stage Cancer of Rectum OR early stage Rectum Cancers OR early stage Rectal Cancer OR early stage Cancer, Rectal OR early stage Rectal Cancers OR early stage Rectum Cancer OR early stage Cancer, Rectum OR early stage Cancer of the Rectum OR early Rectal Neoplasms OR early Neoplasm, Rectal OR early Rectal Neoplasm OR early Rectum Neoplasms OR early Neoplasm, Rectum OR early Rectum Neoplasm OR early Rectal Tumors OR early Rectal Tumor OR early Tumor, Rectal OR early Neoplasms, Rectal OR early Cancer of Rectum OR early Rectum Cancers OR early Rectal Cancer OR early Cancer, Rectal OR early Rectal Cancers OR early Rectum Cancer OR early Cancer, Rectum OR early Cancer of the Rectum Transanal Endoscopic Microsurgery OR Endoscopic Microsurgeries, Transanal OR Endoscopic Microsurgery, Transanal OR Microsurgeries, Transanal Endoscopic OR Microsurgery, Transanal Endoscopic OR Transanal Endoscopic Microsurgeries Radical surgery OR radical excision OR radical resection OR total mesorectal excision OR abdominoperineal resection OR major resection Clinical study OR Clinical trial OR Observational study OR Randomized controlled trial OR Randomized OR RCT					

### Inclusion and exclusion criteria

#### Inclusion criteria

The inclusion criteria were as follows:

#### Exclusion criteria

The exclusion criteria were as follows:

1. Patients with non-rectal cancer or higher stage of rectal cancer (UICC II–IV)
2. Any other interventions or comparators or absence of interventions
3. Literature in other languages
4. Lack of sufficient data or results
5. Duplicate publications
6. In vitro experiments, animal experiments, non-comparative studies, reviews, letters, guidelines, case reports, pathological mechanisms, conference abstracts, expert opinions, editorials, and comments
7. Full text not available

### Data extraction

According to the inclusion and exclusion criteria, two reviewers independently extracted relevant data from the included publications using a standard data extraction form, and the final extraction results were determined through discussions. The outcome measures extracted from the literatures were as follows:

#### Study characteristics

The study characteristics were as follows: first author, year of publication, country, type of trial, study period, and sample size.

#### Patient baseline

The patient baseline was as follows: intervention mode, preoperative T stage, preoperative therapy, age (year old), gender (male/female), R0 resection (%), histological grade, distance from the anus, lymph node metastasis, and follow-up time.

#### Study outcomes

The study outcomes were the following: operation time, blood loss, time of hospitalization, permanent stoma, temporary stoma, postoperative complications, postoperative bleeding, anastomosis stenosis, dehiscence of the suture line or anastomosis leakage, pneumonia, and rectal pain.

#### Follow-up outcomes

The follow-up outcomes were local recurrence, distant metastasis, overall recurrence, disease-free survival, disease-specific survival, perioperative mortality, and overall survival.

### Quality assessment

Two investigators used the Newcastle–Ottawa scale (NOS) [13] to independently evaluate cohort studies. The NOS evaluation scale consists of three aspects: cohort selection (4 stars), comparability between RS and TEM groups (2 stars), and outcome assessment (3 stars), for a total of 9 stars. One to 3 stars were considered as low-quality studies, 4 to 6 stars were considered as moderate-quality studies, and 7 to 9 stars were considered as high-quality studies. Similarly, two investigators used the Cochrane Risk of Bias Assessment System (Review Manager 5.4 tool) to evaluate randomized controlled studies. The assessment system includes six aspects: random allocation method, allocation concealment, blinding, completeness of outcome data, selective reporting of study results, and other sources of bias. Each evaluation aspect can be rated at three levels: low risk, high risk, and unclear risk. Disagreements between two investigators on the evaluation of the literatures will be resolved by negotiation or by having a third-party expert review.

### Statistical analysis

Different methods were used to conduct meta-analysis of effect sizes according to the type of effect size. For dichotomous variables, when sample size and outcome event rates were available in the original literature, the analysis could be performed directly with statistical software; when sample size and outcome event rates were not available in the original literature, the analysis could be performed with RR values and 95% confidence intervals. For continuous variables, the mean values and standard deviations were obtained from the literature for analysis; when continuous variables were presented as median and interquartile spacing, conversions could be performed with an online calculator provided by professor Tiejun Tong, Department of Mathematics, the University of Hong Kong, at the following links: hhttp://www.math.hkbu.edu.hk/~tongt/papers/median.html. *I*^2^ statistic was used to evaluate the heterogeneity between different literatures. When *I*^2^ > 50%, indicating obvious heterogeneity, a random effects model was used for data analysis. When* I*^2^ > 50%, indicating weak heterogeneity, a fixed-effect model was used for data analysis. This meta-analysis used Review Manager 5.4 and Stata MP 17. *p* values < 0.05 were considered statistically significant differences.

## Results

### Literature search results

The search formula was developed to search in four English databases: 101 articles in PubMed, 54 articles in Web of Science, 29 articles in Cochrane library, and 13 articles in Embase. From other meta-analysis/reviews [10, 14] were 13, and the total number of literatures was 211. Duplicates were excluded, leaving 170 articles. According to the inclusion and exclusion criteria, 35 reviews, 5 conference abstracts, 54 inconsistent interventions, and 60 irrelevant studies were excluded by reading the titles and abstracts of the literatures. Sixteen full-text papers were read, and one full-text paper, one case report, and one Russian paper were excluded. Finally, this meta-analysis included 13 literatures, including 5 randomized controlled studies and 8 cohort studies.

### Basic characteristics of the included studies

Thirteen papers, including 5 randomized controlled studies and 8 cohort studies, were included in this meta-analysis, and specific information is provided in Table 2.

**Table 2:** Characteristics of included studies

Study	Year	Type of trial	Study period	Intervention		Preoperative T stage	Preoperative therapy		Age (year old)		Sample size		Gender (male/female)	
				Group A	Group B		Group A	Group B	Group A	Group B	Group A	Group B	Group A	Group B
Heintz et al. [20]	1998	RC	1985 ~ 1996	TEM	RS	T_1_	None	None	66 ± 10	64 ± 10	46	34	NR	NR
Langer et al. [10]	2003	RC	1990 ~ 2001	RS	TEM	T_1_	None	None	66.6 ± 10.5, 62.1 ± 11.1	67.3 ± 12.7	79	27	44/25	15/12
Lee et al. [21]	2003	RC	1994 ~ 2000	TEM	RS	T_1~2_	None	None	61.1 ± 11.2	57.7 ± 11.8	74	100	37/37	51/49
Ptok et al. [ 22]	2007	RC	2000 ~ 2004	TEM	RR	T_1_	None	None	67.2 (95%CI, 64.0 ~ 70.3)	66.3(95%CI, 65.3 ~ 67.9)	35	359	20/15	200/159
Palma et al. [11]	2009	RC	1998 ~ 2005	TEM	RS	T_1_	None	None	68.4 ± 10.7	65.6 ± 9.7	34	17	NR	NR
Stornes et al. [25]	2016	RC	2000 ~ 2009	TEM	TME	T_1~2_	None	None	75.2	69.5	94	2042	51/43	1127/915
De Graaf et al. [16]	2009	PC	NR	TEM	TME	T_1_	None	None	71 (44 ~ 92)	67 (48 ~ 83)	80	75	48/32	48/27
Allaix et al. [15]	2012	PC	1999 ~ 2009	TEM	LR	T_2_	Part of patients from TEM group received neoadjuvant radiotherapy	None	72 (38–91)	65 (34–90)	41	43	16/25	16/19
Winde et al. [19]	1996	RCT	1984 ~ 1992	TEM	AR	T_1_	None	None	63.7 (36 ~ 90)	60.9 (47 ~ 81)	24	26	10/14	14/12
Lezoch et al. [18]	2012	RCT	1997 ~ 2004	ELRR by TEM	TME	T_2_	nCRT	nCRT	66 (58 ~ 70)	66 (60 ~ 69)	50	50	30/20	34/16
Chen et al. [9]	2013	RCT	2008 ~ 2010	TEMS	LAR	T_1~2_	None	None	68.8 ± 5.3	66.2 ± 7.7	30	30	14/16	17/13
Bach et al. [23]	2021	RCT	2012 ~ 2014	TEM	TME	T_1~3_	Neoadjuvant radiotherapy	None	RCT, 65(52 ~ 79) NRCT, 74(53 ~ 89)	RCT, 65 (49 ~ 83) NRCT, 69 (53 ~ 72)	RCT, 27 NRCT, 61	RCT, 28 NRCT, 7	RCT, 19/8 NRCT, 39/22	RCT, 17/11 NRCT, 4/3
Lezoche et al. [17]	2008	RCT	NR	TEM	LR	T_2_	nCRT	nCRT	67 (61 ~ 70)	65 (60 ~ 69)	35	35	12/23	15/20

Study	Country	R0 resection (%)		Histological grade I /II: yes or no	Distance from the anus(cm)		Lymph node metastasis	Follow-up(month)	
		Group A	Group B		Group A	Group B		Group A	Group B
Heintz et al. [20]	Germany	75.1	100	Low-risk group: yes High-risk group: no	NR	NR	Low-risk group: N0 High-risk group: Part of patients was N +	Low-risk group: 52 ± 22.7 High-risk group: 42.8 ± 21.6	Low-risk group: 52 ± 22.7 High-risk group: 42.8 ± 21.6
Langer et al. [10]	Germany	NR	NR	Yes	9.5 ± 3.9	7.7 ± 2.3 6 ± 3.2	N0	NR	NR
Lee et al. [21]	Korea	100	100	Yes	NR	NR	N0	31.0 ± 17.2	34.6 ± 19.4
Ptok et al. [22]	American	100	100	Yes	NR	NR	N0	42.7 (39.7 ~ 45.8)	42.4 (40.8 ~ 44.0)
Palma et al. [11]	Germany	91.18	100	Yes	10.9 ± 3.5	8.9 ± 3.2	N0	93.0 (48 ~ 108)	86.5 (48 ~ 113)
Stornes et al. [25]	Norway	T_1_, 89 T_2_, 86	T_1_, 98 T_2_, 98	No	3.66	8.67	N0	NR	NR
De Graaf et al. [16]	Netherlands	94.2	98.7	No	8.0(0 ~ 15)	7.0(0 ~ 15)	N0	NR	NR
Allaix et al. [15]	Italy	90.2	100	No	6(3 ~ 11)	5(1 ~ 12)	Part of patients was N +	NR	NR
Winde et al. [19]	Germany	NR	NR	Yes	NR	NR	N0	40.9 ± 24.6	45.8 ± 24.6
Lezoch et al. [18]	Italy	100	100	Yes	4.92(3 ~ 6)	5(3 ~ 6)	N0	115.2 ± 20.64	115.2 ± 22.8
Chen et al. [9]	China	100	100	Yes	7.8 ± 1.6	8.1 ± 2.3		18.0 ± 2.6	17.5 ± 2.2
Bach et al. [23]	UK	RCT, 85 NRCT, 82	RCT, 93 NRCT, 100	Part of patients was No	RCT, 6 (4 ~ 8) NRCT, 6(4 ~ 7)	RCT, 6 (5 ~ 7) NRCT, 5.5 (3.5 ~ 7.5)	Part of patients was N +	RCT: 4.28 years (IQR3.27 ~ 5.02) NRCT: 4.07 years (IQR 3.19 ~ 4.69)	RCT: 4.28 years (IQR3.27 ~ 5.02) NRCT: 4.07 years (IQR 3.19 ~ 4.69)
Lezoche et al. [17	Italy	NR	NR	YES	< 6	< 6	N0	84 (71 ~ 97)	84 (76 ~ 96)

*TEM* transanal endoscopic microsurgery, *LR* laparoscopic resection, *AR* anterior resection, *RS* radical surgery, *RR* radical resection, *TME* total mesorectal excision, *ELRR* endoluminal locoregional resection, *nCRT* neoadjuvant chemoradiotherapy, *LAR* laparoscopic lower anterior resection, *NR* not reported

### Results of meta-analysis

#### Operative time

Seven papers [9, 11, 15–19] reported the operative time (minute) of both TEM and RS; there was significant heterogeneity (*I*^2^ = 93% > 50%, and *Q* test *P* < 0.05); no source of heterogeneity was found, so meta-analysis was performed based on random effects, and the results were as follows: the result after meta-combination was −97.14 (−115.81, −78.47), and there was a significant difference between TEM and RS (*Z* = 10.20, *P* < 0.05) (see Fig. 3A). On average, the operative time for TEM was 97.14 min shorter than that for RS, which means that the TEM surgical approach was effective in reducing the operative time Table 3.

**Table 3:** NOS quality evaluation

Study	Design	Selection		Comparability	Outcome	Total
Heintz et al. [20]	RC	★★★★	★		★★★★★	8
Langer et al. [10]	RC	★★★★★	★		★★★★	6
Lee et al. [21]	RC	★★★★★	★		★★★★★	7
Ptok et al. [22]	RC	★★★★★	★★★★		★★★★	7
Palma et al. [11]	RC	★★★★★	★		★★★★★	7
Stornes et al. [24]	RC	★★★★	★		★★★★★★	8
De Graaf et al. [16]	PC	★★★★★★	★★★★		★★★★★★	8
Allaix et al. [15]	PC	★★★★★★	★		★★★★	6

NOS quality assessment items: selection, (1) representativeness of the exposed cohort. (2) representative of the non-exposed cohort. (3) determination of exposure. (4) No subjects had developed the disease under study at the start of the study. Comparability, exposed cohort and non-exposed cohort. Comparability of cohorts; outcomes, (1) outcome measurement method. (2) Whether follow-up time was long enough. (3) Follow-up completeness

#### Blood loss

Seven papers [9, 11, 15–19] reported surgical bleeding (ml) for both TEM and RS with significant heterogeneity (*I*^2^ = 99% > 50%,and *Q* test *P* < 0.05). By rejecting each article, no source of heterogeneity was found, so a meta-analysis was performed based on random effects, and the results were as follows: the effect size after meta-combination was −315.52 (−472.47, −158.57), and there was a significant difference between TEM and RS (*Z* = 3.94, *P* < 0.05) (see Fig. 3B).The surgical bleeding volume of TEM was 315.52 ml less than that of RS, which means that the TEM surgical approach was effective in reducing surgical bleeding volume.

#### Time of hospitalization

Six papers [9–11, 15, 18, 19] reported length of stay (days) for both TEM and RS procedures with significant heterogeneity (*I*^2^ = 82% > 50%, and *Q* test *P* < 0.05), and by excluding the literature one by one, Lezoche et al. [17] was found to be one of the sources of heterogeneity. Excluding Lezoche et al. [17], for meta-analysis again, the heterogeneity was significantly reduced (*I*^2^ = 51% > 50%, and *Q* test* P* > 0.05). So meta-analysis was performed based on random effects, and the results were as follows: the result after meta-merge was −8.82 (−10.62, −7.62), and there was a significant difference between TEM and RS (*Z* = 11.05, *P* < 0.05) (see Fig. 3C), and the length of stay was reduced by 8.82 days in case of TEM compared with RS, which means that the TEM surgical approach was significant in reducing the length of stay.

#### Postoperative complication

**Fig. 4:**
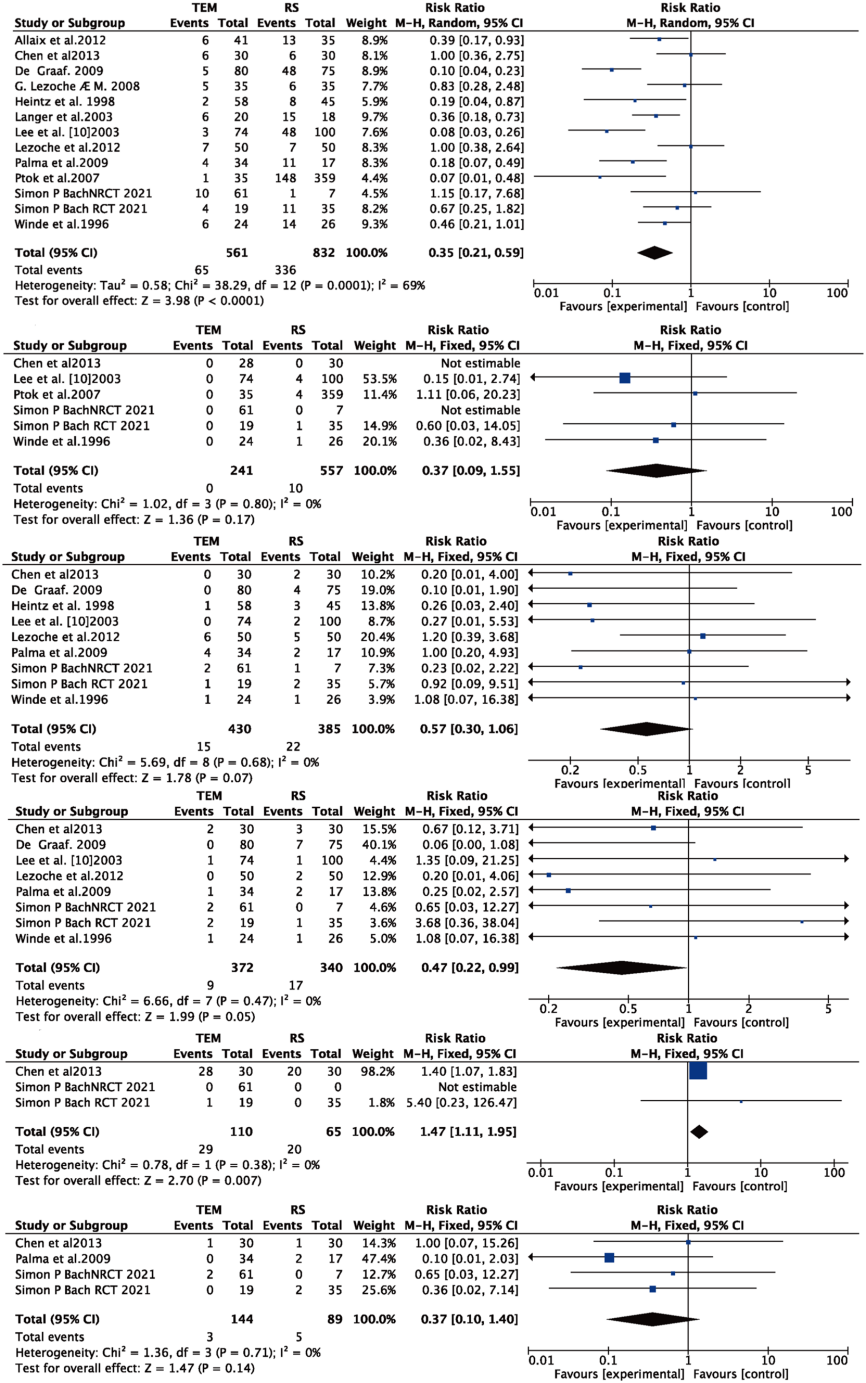
Forest plot: **A** postoperative complication, **B** anastomosis stenosis, **C** dehiscence of the sutureline or anastomosis leak, **D** postoperative bleeding, **E** rectal pain, **F** pneumonia

Twelve papers [9–11, 15–23] reported postoperative complications for both TEM and RS procedures with significant heterogeneity (*I*^2^ = 69% > 50%, and *Q* test *P* < 0.05), and no source of heterogeneity was found by article-by-article elimination, so a meta-analysis based on random effects was performed with the following results: the result after meta-combination was 0.35 (0.21, 0.59), and there was a significant difference between TEM and RS (*Z* = 3.98, *P* < 0.05) (see Fig. 4A), and the postoperative complication rate of TEM was lower than that of RS, showing that the TEM surgical approach was significantly effective in reducing the postoperative complication rate. Four papers [19, 21–23] reported the incidence of postoperative anastomotic stenosis for both TEM and RS procedures without significant heterogeneity (*I*^2^ = 0% < 50%, and *Q* test *P* > 0.05), so a meta-analysis based on fixed effects was performed with the following results: the result after meta-combination was 0.37 (0.09, 1.55), but there was no significant difference between TEM and RS (*Z* = 1.36, *P* > 0.05) (see Fig. 4B). Eight papers [9, 11, 16, 18–23] reported the incidence of postoperative suture dehiscence or anastomotic leak for both TEM and RS procedures without significant heterogeneity (*I*^2^ = 0% < 50%,and *Q* test *P* > 0.05), so a meta-analysis based on fixed effects was performed with the following results: the result after meta-combination was 0.57 (0.30, 1.06). However, there was no significant difference in the incidence of suture dehiscence or anastomotic leakage between the two surgical approaches (*Z* = 1.78, *P* > 0.05) (see Fig. 4C). Seven papers [9, 11, 16, 18, 19, 21, 23] reported the incidence of postoperative bleeding for both TEM and RS procedures. There was no significant heterogeneity (*I*^2^ = 0% < 50%, and *Q* test *P* > 0.05), so a meta-analysis based on fixed effects was performed with the following results: the result after meta-combination was 0.47 (0.22, 0.99), but there was no significant difference in the incidence of postoperative bleeding between the two surgical approaches (*Z* = 1.99, *P* = 0.05) (see Fig. 4D). Two papers [9, 23] reported the incidence of postoperative rectal pain after both TEM and RS procedures without significant heterogeneity (*I*^2^ = 0% < 50%, and *Q* test *P* > 0.05), so a meta-analysis based on fixed effects was performed, and the results were as follows: the result after meta-combination was 1.47 (1.11, 1.95). The incidence of rectal pain after TEM was 1.47 time higher than that of RS; there was a significantly different in the incidence of postoperative rectal pain between the two surgical approaches (*Z* = 2.70, *P* < 0.05) (see Fig. 4E). Three papers [9, 11, 23] reported the incidence of pneumonia after both TEM and RS procedures without significant heterogeneity (*I*^2^ = 0% < 50%, and *Q* test *P* > 0.05), so a meta-analysis based on fixed effects was performed, and the results were as follows: the result after meta-combination was 0.37 (0.10, 1.40), and the incidence of pneumonia after TEM was 0.37 times higher than the incidence of pneumonia after RS, but there was no significant difference in the incidence of postoperative pneumonia between the two surgical approaches (*Z* = 1.47, *P* > 0.05) (see Fig. 4F).

### Temporary stoma

**Fig. 5:**
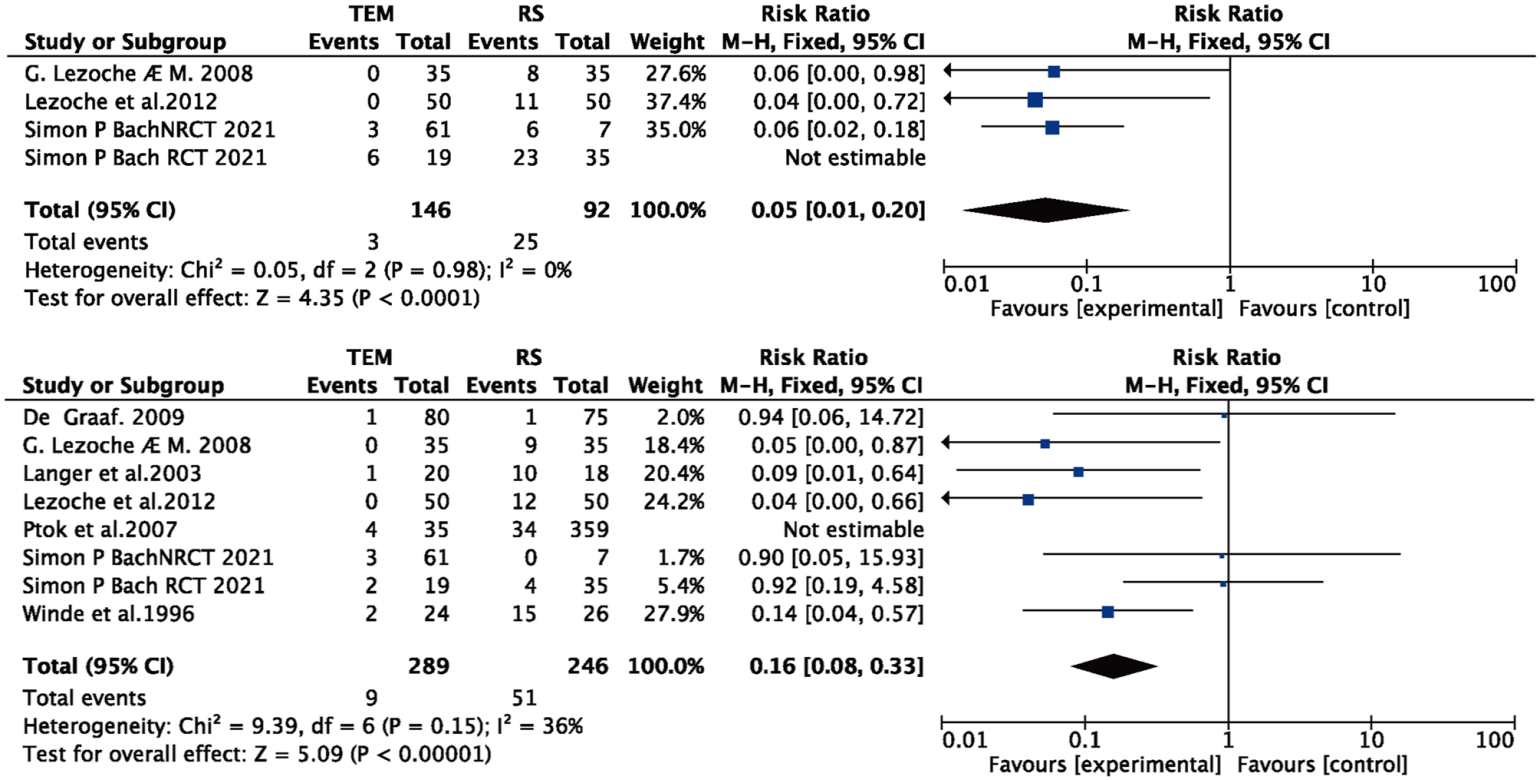
Forest plot: **A** temporary stoma, **B** permanent stoma

Three papers [17, 18, 23] reported temporary stoma for both TEM and RS, with significant heterogeneity (*I*^2^ = 76% > 50%, and *Q* test *P* < 0.05). By excluding each article, Simon P Bach (2021) [23] was found to be the source of heterogeneity. After excluding this article (*I*^2^ = 0% > 50%, and *Q* test *P* > 0.05), meta-analysis based on fixed effects was performed, and the results were as follows: the result after meta-merger was 0.05 (0.01, 0.20), and there was a significant difference between TEM and RS (*Z* = 4.35, *P* < 0.05) (see Fig. 5A); the temporary stoma of TEM was lower than that of RS.

### Permanent stoma

Seven papers [10, 16–19, 22, 23] reported permanent stoma for both TEM and RS with significant heterogeneity (*I*^2^ = 61% > 50%,and *Q* test *P* < 0.05), and by excluding each article, Ptok was found to be the source of heterogeneity, and after excluding this article, *I*^2^ = 36% < 50%, and *Q* test *P* > 0.05, so meta-analysis based on fixed effects was performed: the result after meta-merge was 0.16 (0.08, 0.33), and there was a significant difference between TEM and RS (*Z* = 5.09, *P* < 0.05) (see Fig. 5B); the permanent stoma of TEM was lower than that of RS.

### Perioperative mortality

**Fig. 6:**
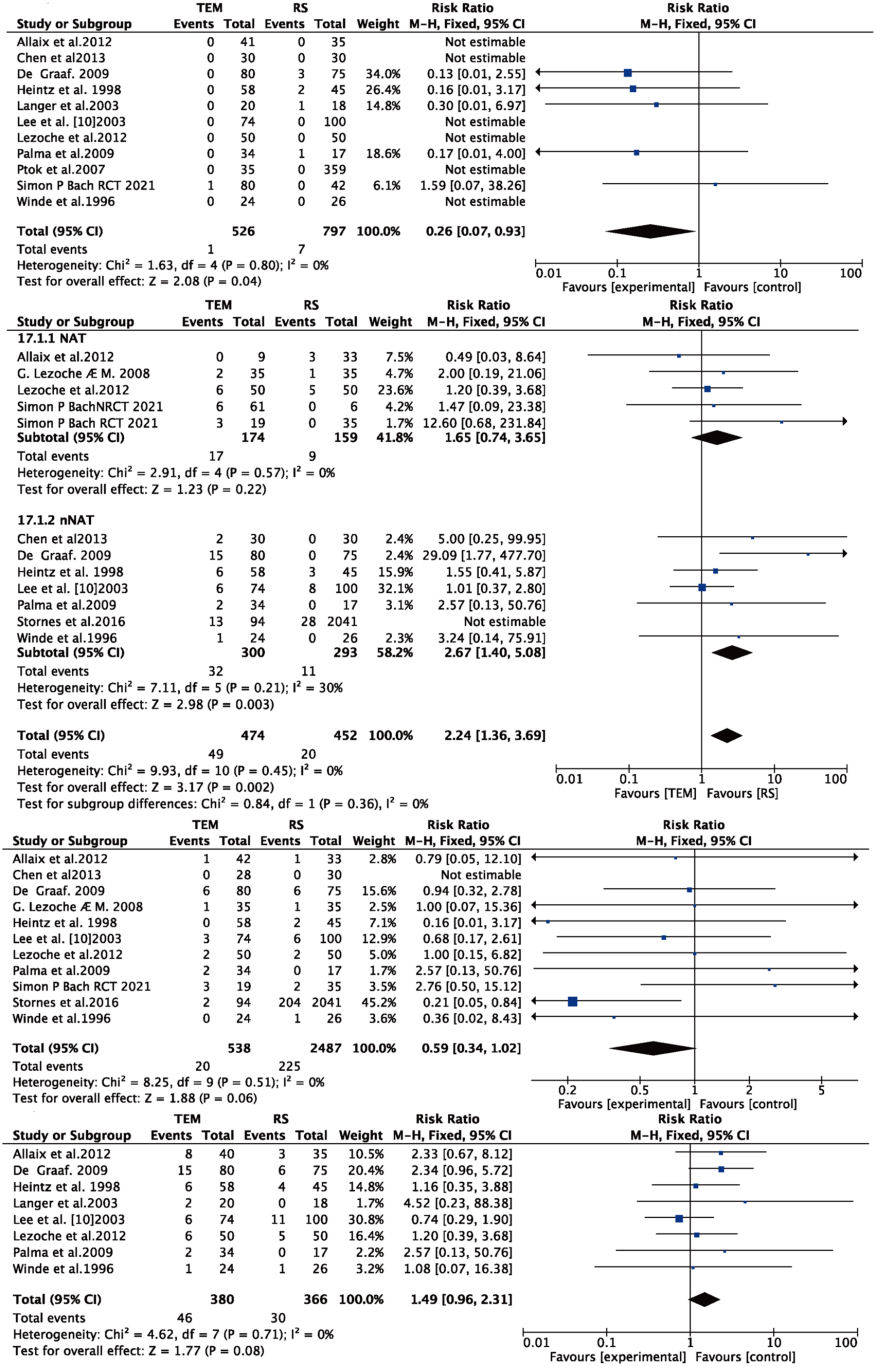
Forest plot: **A** Perioperative mortality, **B** local recurrence (included articles in which patients in the transanal endoscopic microsurgery (TEM) group underwent neoadjuvant therapy), **C** distant metastasis, **D** overall recurrence

Eleven papers [9–16, 18–23] reported perioperative mortality for both TEM and RS with no significant heterogeneity (*I*^2^ = 0% < 50%, and *Q* test *P* > 0.05), so a meta-analysis based on fixed effects was performed with the following results: the result after meta-combination was 0.26 (0.07, 0.93), and there was a significant difference between TEM and RS (*Z* = 2.08, *P* < 0.05) (see Fig. 6A). The perioperative mortality for TEM was 0.26 time greater than that for RS.

### Local recurrence

Eleven publications [9, 11, 15–24] reported local recurrence rates for both TEM and RS procedures with significant heterogeneity (*I*^2^ = 55% > 50%, and *Q* test *P* < 0.05); by excluding the literature one by one, Stornes [24] was found to be the source of heterogeneity. By excluding this literature (*I*^2^ = 0% < 50%, and *Q* test *P* > 0.05), therefore based on fixed effects, a meta-analysis was performed, and the results were as follows: the result after meta-combination was 2.51 (1.53, 4.21), and there was a significant difference between TEM and RS (*Z* = 3.65, *P* < 0.05); the local recurrence rate for TEM was 2.51 times higher than the local recurrence rate for RS. Figure 6 is a subgroup analysis based on whether neoadjuvant treatment was performed. Intervention of three papers [14, 17, 22] was TEM with neoadjuvant therapy and RS, and the other literature[16] was neoadjuvant therapy for both TEM and RS. The results showed that with neoadjuvant therapy, there was no significant difference in local recurrence rate for both procedures (*Z* = 1.23, *P* > 0.05). While without neoadjuvant therapy, TEM was significantly higher than RS regarding the local recurrence rate (*Z* = 2.98, *P* < 0.05).

### Distant metastasis

Eleven papers [9, 11, 15–21, 23, 24] reported the incidence of distant metastasis for both TEM and RS surgical procedures without significant heterogeneity (*I*^2^ = 0% < 50%, and *Q* test *P* > 0.05), so a meta-analysis based on fixed effects was performed with the following results: the result after meta-combination was 0.59 (0.34, 1.02), and the distant metastasis for TEM was lower than that for RS. However, there was no significant difference in distant metastasis between the two surgical approaches of TEM and RS (see Fig. 6C).

### Overall recurrence

Eight papers [10, 11, 15, 16, 18–21] reported overall recurrence after both TEM and RS procedures without significant heterogeneity (*I*^2^ = 0% < 50%, and *Q* test *P* > 0.05), so a meta-analysis based on fixed effects was performed, and the results were as follows: the result after meta-combination was 1.49 (0.96, 2.31), and the overall recurrence rate for TEM was 1.49 times higher than that for RS, but there was no significant difference in overall recurrence between the two surgical modalities of TEM and RS (see Fig. 6D).

### Overall survival

**Fig. 7:**
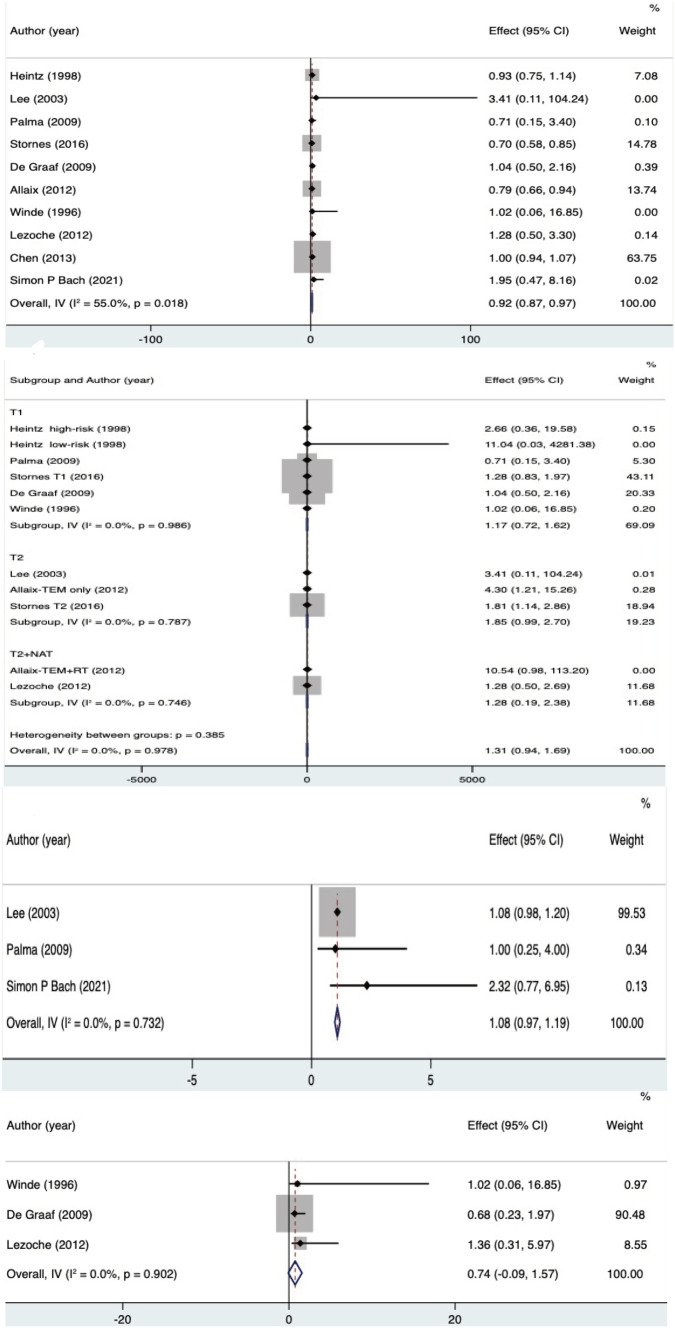
**A** Overall survival, **B** overall survival (subgroup of T_1_, T_2_, T_2_ combined with neoadjuvant therapy), **C** disease-free survival, **D** disease-specific survival

Ten publications [9, 11, 15, 16, 18–21, 23, 24] reported overall survival after both TEM and RS procedures. And the effect size was the RR value and 95% CI of overall survival. There was significant heterogeneity (*I*^2^ = 55% > 50%, and *Q* test *P* < 0.05). No source of heterogeneity was found by article-by-article elimination, so a meta-analysis was performed based on random effects, and the results were as follows: the result after meta-combination was 0.88 (0.74, 1.00), and the effect size was significant difference (*Z* = 12.92, *P* < 0.05) (see Fig. 7A). The overall survival rate for TEM was lower than the overall survival rate for RS. The results of the subgroup analysis showed that the effect values were hazard ratio and 95% confidence interval; TEM reduced overall survival (HR = 1.314 (95% CI 0.931, 1.697)) with significant difference (*Z* = 6.721, *P* = 0.000 < 0.05); for the T_2_ early-stage cancer without neoadjuvant treatment subgroup [15, 21, 24], the adverse effect of TEM on overall survival was more significant (HR = 1.847 (95% CI 0.994, 2.701)), with statistically significant (*Z* = 4.242, *P* = 0.000 < 0.05). However, for the T_2_ early-stage cancer, combined with neoadjuvant therapy subgroup [15, 18] (HR = 1.286 (95% CI −0.114, 2.685)), TEM did not significantly reduce overall survival (see Fig. 7B).

### Disease-free survival

Three papers [11, 21, 23] reported disease-free survival after both TEM and RS procedures. The effect value was RR values for disease-free survival and 95% CI. Without no significant heterogeneity (*I*^2^ = 0% < 50%, and *Q* test *P* > 0.05), meta-analysis-based fixed effects was performed. The results were as follows: the result after meta-combination was 1.08 (0.97, 1.19), and the effect size was significant (*Z* = 19.31, *P* < 0.05) (see Fig. 7C). Disease-free survival surgery for TEM was 1.08 times higher than disease-free survival surgery for RS; that is, both TEM and RS surgical modalities differed significantly in terms of disease-free survival.

### Disease-specific survival

Three papers [16, 18, 19] reported disease-specific survival after both TEM and RS; effect sizes were RR values and 95% CI for overall survival without significant heterogeneity (*I*^2^ = 0% < 50%, and *Q* test *P* > 0.05), so meta-analysis based on fixed effects was performed with the following results: the result after meta-combination were 0.74 (0, 091.54), *Z* = 1.756 *P* = 0.079 > 0.05 (see Fig. 7D), and the disease-free survival surgery of TEM was 0.74 times higher than the surgical disease-free survival of RS, but there was no significant difference for both TEM and RS.

## Discussion

Based on the results of this meta-analysis, TEM was associated with a higher risk of local recurrence compared with RS and was superior in terms of disease-free survival, reduced perioperative mortality, temporary stoma, permanent stoma, and postoperative complications; however, the overall survival of TEM was lower than RS, and the rectal pain rate was higher than RS. In addition, TEM was effective in reducing operative time, postoperative bleeding, and time of hospitalization. There was no statistically significant difference between the two surgical approaches in terms of distant metastasis, overall recurrence, disease-specific survival, and the incidence of postoperative complications such as suture dehiscence or anastomotic leakage, postoperative bleeding, pneumonia, and anastomotic stenosis. Our findings demonstrate that TEM is one of the effective and safe alternatives to RS, and more high-quality studies are needed to prove this.

In clinical practice, local recurrence is a complication to avoid with regard to the choice of TEM. We concluded that the local recurrence rate was higher in patients treated with TEM compared with RS, while the overall recurrence rate was no significantly different. After rigorous and careful screening, patients who experienced recurrence can undergo salvage surgery again. However, 5-year overall survival rate was 50% (95% CI, 30–74%), and re-recurrence-free survival was 47% (95% CI, 25–68%). Oncologic outcomes are poor[26]. Several studies have come to the same conclusion [14, 27]. Possible reasons for high local recurrence after TEM are as follows: high-grade pathological grading, distance of the incision margin from the margin of the primary focus, T-staging, and occult lymph node metastasis. The study of T Junginger [28] showed that the probability of local recurrence was 11% for low-grade pathology and 25% for high-grade pathology and 10% and 38% for local recurrence when the pathology was both low risks, and the distance of the cut edge from the edge of the primary focus was > 1 mm and < 1 mm, respectively. The study of Morino [29] showed that sm stage, T stage, and tumor grade were independent predictors of local recurrence in multivariate analysis. When no intervention was taken for local recurrence, the median survival of patients was at 8 months [30]. The median survival of patients with rectal cancer recurrence is 47%, 38%, and 35% at 5, 10, and 15 years, respectively [31, 32]. Although salvage RS can remove recurrent lesions after local recurrence in patients who have undergone TEM, most patients do not achieve complete resection [33]. Therefore, RS has a better outcome in terms of survival. TEM combined with neoadjuvant therapy is similar to RS in terms of local recurrence and survival in early-stage rectal cancer (T_2_), and TEM is one of the alternatives to RS in appropriately selected patients [34].

In terms of quality of life, we concluded that the rate of stoma is significantly higher in both temporary and permanent stoma in RS than in TEM. Postoperative stoma can significantly impair physiological function and reduce quality of life [35]. The extensive and invasive nature of RS not only significantly impairs patients’ quality of life and social functioning, but also promotes health anxiety and affects family social relationships. Simon P Bach [23] has shown that RS is associated with adverse symptoms, such as changes in bowel habits, urinary incontinence, bowel dysfunction, and sexual dysfunction, which may have a negative impact on daily life. On the contrary, TEM is a local excisional surgery, which can preserve the autonomic nerves in the pelvic cavity, thus preserving the normal urinary, bowel, and sexual functions; at the same time, it protects the anal sphincter muscle, thus preserving the anal function and reducing the probability of stoma. Our study found that the TEM surgical approach significantly reduced the overall incidence of postoperative complications, but there were no significant differences between two surgical approaches. One study [14] concluded that TEM was more advantageous in terms of overall postoperative complication rates and that the TEM reduced both anastomotic stenosis and postoperative bleeding. The reason for the different results may be that a randomized controlled study included in our study [23] intervention was that short-term radiotherapy combined with TEM had a positive impact on the occurrence of postoperative complications. Given that TEM can reduce the incidence of postoperative complications and improve quality of life, this surgical approach is suitable for elderly patients with severe diseases, poor physical fitness, and short life expectancy, and the TEM surgical approach can significantly reduce the operative time, intraoperative bleeding, and length of hospital stay and reduce the financial burden on patients. The results of this study showed that the perioperative mortality was lower with the TEM approach, which was 0.26 times lower than that of RS. E.J.R. De Graaf [16] study showed that the perioperative mortality rate for RS ranged from 3.3 to 25.8%. Also, some studies [36, 37] showed that TEM has been a safer surgical procedure with a clear view of the surgical field, clear identification of tissue structures, precise separation of the tumor, less stoma, and fewer complications than RS. In terms of long-term survival, we concluded that the TEM surgical approach reduced overall survival, and the Xiaoyu Xiong [14] found that similar results were obtained in the study, where the adverse effect of TEM on overall survival was more pronounced when subgroup analysis was performed with T_2_ staging and without neoadjuvant therapy; when neoadjuvant therapy was performed to patients with early-stage rectal cancer, there was no significant difference between the two surgical approaches in terms of overall survival. The results suggest that for rectal cancer patients with T2 stage, neoadjuvant therapy improves overall survival. Intervention of Simon P Bach [23] for early-stage rectal cancer was short-term radiotherapy combined with TEM and TME, and the results showed no significant difference in overall survival between the two interventions over a mean follow-up period of 4.28 years. The results demonstrate that neoadjuvant therapy improves the overall survival of TEM surgery. Our study found that in terms of disease-free survival, although there was a statistically significant difference between TEM and RS, the RR value was only 1.08. This indicated that there was no significant difference between TEM and RS. Tan S [38]. showed that there was no significant difference between local resection and radical resection in terms of disease-free survival. In this respect, TEM or combined neoadjuvant therapy is an alternative option for the treatment of early rectal cancer. Several studies_[39, 40] found that 89% of T_1_ and 72% of T_2_ of rectal cancer patients experienced unnecessary RS procedure, respectively, resulting in a significant increase in the incidence of adverse events such as urinary incontinence, sexual dysfunction, bowel dysfunction, anastomotic leak, and permanent or temporary stoma, which seriously affects the quality of life of patients. Endoscopic and pathologic examination for diagnosis and pathologic grading, endoscopic ultrasound to determine the depth of infiltration, MRI and CT to determine the presence of distant metastases, and screening of patients eligible for surgery are top priorities. According to the 2018 NCCN Guidelines [41], the surgical approach of local excision is recommended for patients with T_1_ and N_0_ with early-stage rectal cancer and tumor diameter < 3 cm, intermediate to advanced pathological grade, and tumor invasion < 30% of the circumference of the bowel, and RS is recommended for patients with T_2_ rectal cancer; T_1_ rectal cancer presenting with positive margins, lymphovascular infiltration, and poorly differentiated or infiltrated to the lower third of the submucosa (sm3 level), RS is recommended [42, 43]. The use of TEM for rectal cancer patients with T_1_ N_0_ and without high-risk pathologic features has received wide acceptance. In these patients, the local recurrence and overall survival rates of TEM are comparable to those of RS and have a much lower impact on quality of life [44]. S. V. Chernyshovf [45] found that the 3-year disease-free survival rates for both TEM and RS were 92% and 96%, respectively, indicating that TEM is a safe and effective surgical procedure for the treatment of early rectal cancer in T1. Other studies [34, 46] found that for patients with T2-staged rectal cancer, local excision alone significantly reduces patient disease-free survival; and when neoadjuvant therapy is combined with local excision, the oncologic outcome can be compared to that of RS. Neoadjuvant therapy combined with local excision [47–49] can be applied to T2 and even to T3 and T4, and the neoadjuvant therapy can reduce the tumor stage and improve the organ preservation rate of the rectum in order to improve the quality of life while obtaining good tumor outcome. Neoadjuvant chemotherapy has been shown to improve local recurrence rates and reduce toxicity. However, it is unclear that the impact of neoadjuvant therapy on survival in patients with early-stage rectal cancer who undergo radical surgery adhering to total mesorectal excision [50].

Meanwhile, this meta-analysis has some limitations. Firstly, most of the included literatures were non-randomized controlled studies, and the reliability of the conclusions was low; secondly, the inclusion criteria of study subjects were different among different literatures, and there was a strong heterogeneity; third the outcome index measures of different literatures had a large heterogeneity, which might lead to less reliable conclusions. In the future, we will conduct prospective, multicenter, randomized controlled studies with large samples to provide a reliable basis for clinical decision-making.

## Conclusion

In conclusion, limited evidence suggests that TEM can shorten operative time and hospital stay, reduce intraoperative bleeding, decrease the incidence of postoperative complications and perioperative mortality, reduce stoma rates, and improve quality of life and is a safe surgical alternative to RS in selected patients. Neoadjuvant therapy in combination with TEM is promising to improve the higher local recurrence rate of TEM and long-term survival and could be tried in patients with advanced rectal cancer.

## Data Availability

Data available on request from the authors.
